# The Open Syndrome Definition as a Machine-Readable Standard for Public Health: Design and Implementation Study

**DOI:** 10.2196/86249

**Published:** 2026-06-18

**Authors:** Ana Paula Gomes Ferreira, Aleksandar Anžel, Izabel Marcilio, Helen Hughes, Alex J Elliot, Jude Dzevela Kong, Madlen Schranz, Alexander Ullrich, Georges Hattab

**Affiliations:** 1 Center for Artificial Intelligence in Public Health Research, Robert Koch Institute, Nordufer 20, Berlin, D-13353, Germany, +49 30 18754 0, +49 30 18754 2328; 2 Department of Mathematics and Computer Science, Freie Universität Berlin, Berlin, Germany; 3 Center for Data and Knowledge Integration for Health, Fundação Oswaldo Cruz, Salvador, Brazil; 4 Real-time Syndromic Surveillance Team, UK Health Security Agency, Birmingham, United Kingdom; 5 Department of Mathematics and Statistics, Africa-Canada Artificial Intelligence and Data Innovation Consortium, York University, Toronto, ON, Canada; 6 Infectious Disease Epidemiology Department, Robert Koch Institute, Berlin, Germany

**Keywords:** public health surveillance, epidemiological monitoring, health information exchange, public health, AI, data standardization, artificial intelligence

## Abstract

**Background:**

Case definitions are essential for effectively communicating public health threats. However, the absence of a standardized, machine-readable format poses significant challenges to interoperability, epidemiological research, data sharing, and the application of computational methods, including artificial intelligence. These barriers complicate collaboration across regions and organizations and hinder technological progress in public health.

**Objective:**

This study aims to propose and release the first open, machine-readable format for representing case and syndrome definitions, together with tools and resources that enable their standardized and scalable use.

**Methods:**

We developed the Open Syndrome Definition, a structured, machine-readable schema for representing case and syndrome definitions. We compiled official public health case definitions from multiple institutions and converted them into standardized, machine-readable representations using open-source tools. These tools, available through GitHub under the Massachusetts Institute of Technology license, automate the translation of narrative definitions into structured data. We also created a platform for browsing, analyzing, and contributing new definitions on our initiative website.

**Results:**

The Open Syndrome Definition format enabled consistent, automated representation of case definitions across different diseases and jurisdictions. The conversion tools achieved high semantic fidelity, as assessed by qualitative expert review, between narrative and structured representations, supporting human verification and automated analysis. The dataset and accompanying tools demonstrated structural and semantic interoperability by standardizing definitions from various health systems into a unified format and integrating existing medical ontologies through JSON for Linked Data. To further illustrate practical applicability and downstream usage, we introduced a data filtering prototype that allows users to upload their own datasets and verify the results against the standardized definitions.

**Conclusions:**

The Open Syndrome Definition establishes a foundation for consistent and machine-readable public health definitions, facilitating reproducible research and interoperability at scale. By enabling systematic data exchange and artificial intelligence–driven analysis, it strengthens public health preparedness and supports more rapid, coordinated responses to emerging health threats.

## Introduction

Case definitions are essential tools for public health practitioners. They are used to identify, monitor, and respond to diseases or groups of diseases [1,2]. They inform the public, orient public health policies, and guide surveillance indicators, such as syndrome definitions [3]. In the context of public health surveillance, terminology can often be fragmented. While case definitions typically describe strict clinical and laboratory criteria for identifying specific diseases, the criteria used for early warning systems are referred to in the literature as either syndrome definitions or syndromic indicators. Due to the lack of global consensus [4,5], these 2 terms are frequently used interchangeably in practice; therefore, throughout this paper, we treat them as synonymous.

**Figure 1.**
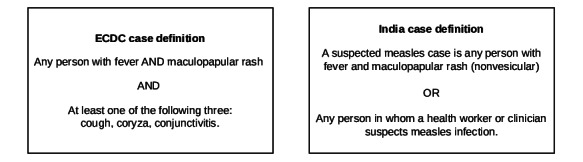
Measles case definition comparison. The definitions from the European Centre for Disease Prevention and Control [6] and India [7] are presented on the left and right, respectively. ECDC: European Centre for Disease Prevention and Control.

Developing case definitions requires expert knowledge of the targeted disease(s), consultation of existing definitions, and analysis of available clinical data. Case definitions are usually written as free-text descriptions of the key characteristics and criteria of the target disease or public health threat. The goal of a case definition is to provide a consistent description for public health officials, health workers, policymakers, and the general public so they can understand a given threat. Figure 1 shows two different case definitions for the same disease from different provenance [6,7]. The lack of interoperability and standardization of syndromic indicators and case definitions makes it difficult to compare systems and epidemiological situations across different regions and time periods [8]. Free-text definitions, in particular, introduce a fundamental gap in translation between human and machine interpretation, resulting in inconsistent and error-prone automated processing [9]. Beyond mere structural inconsistencies, free-text descriptions are highly susceptible to linguistic ambiguity and semantic fragmentation [10]. While complex logical relationships and clinical nuances appear straightforward to trained public health professionals, they become deeply problematic for computational systems.

Consequently, effectively translating these definitions for automated surveillance requires more than just a technical data container; it necessitates a robust methodology to resolve semantic ambiguities and ensure true interoperability across diverse health systems [11]. Examples of these relationships are conditions and nested criteria. These inconsistencies can delay coordinated responses during critical time periods when containing emerging threats is essential. For example, inconsistent case definitions had far-reaching consequences for global disease surveillance and control efforts during the COVID-19 pandemic. The consequences of these issues include underreporting and misclassifications of cases [12], compromised accuracy of surveillance data [13-15], compromised resource allocation [16], and problems comparing disease burden and intervention effectiveness across countries [17]. The variability in case definitions and the absence of structured formats to standardize them represent a surprisingly underexplored gap in public health infrastructure. Moreover, the dearth of structured formats imposes substantial constraints on embracing artificial intelligence (AI) and eventually implementing AI for surveillance and outbreak detection. As health systems increasingly rely on computational approaches, a significant challenge to the use of AI as a technology for protecting public health is posed by the gap between narrative definitions and machine-readable formats. When disease indicators are monitored in real-time or near real-time for the purpose of early outbreak detection, it is imperative to use a structured format for case definitions. This is especially true in the context of syndromic surveillance, where automated data acquisition is used [18].

Earlier studies [19,20] verify that using a structured format for clinical standards in case definitions leads to enhanced reporting accuracy compared to narrative descriptions. These studies emphasize that even well-defined case definitions can be interpreted differently by various users, which can lead to misclassifications, decreased sensitivity, and lower positive predictive value. A highlight from the related work is when case definitions were successfully used to automate the generation of a new case definition and translate an existing one from medical conditions [21]. Notwithstanding this evidence, at the time of writing, no comprehensive effort has been made to develop a standardized, machine-readable format for case definitions. Adopting this format would eliminate any inconsistencies in interpretation and lay the groundwork for the infrastructure needed to support next-generation public health surveillance systems.

As a matter of fact, clearly structured definitions can help AI systems identify diseases and track outbreaks more effectively. This enables machine learning (ML) programs to detect cases more accurately, notice subtle changes in disease patterns, and alert us to emerging health threats, even when traditional symptoms are not yet evident.

To achieve these capabilities, the core scientific contribution of this work is the development of a methodology for semantic interoperability in public health surveillance. As the practical implementation of this methodology, we introduce the Open Syndrome Definition (OSD) framework, a name chosen to reflect its capacity to encompass both case and syndrome definitions. Moving beyond basic structural formatting, our approach leverages a machine-readable representation integrated with semantic web principles (JSON for Linked Data [JSON-LD]) [22,23] and established medical ontologies, successfully bridging the gap between human readability and computational precision. Consequently, OSD offers the flexibility and clarity required to create precise, unambiguous descriptions suitable for AI applications and diverse software tools. Furthermore, by incorporating metadata often missing from traditional narratives, the framework enables the reuse of definitions, cross-jurisdictional comparisons, version control, and downstream applications in ML projects, benchmarks, and publications. Importantly, OSD complements, rather than replaces, traditional narrative structures. By systematically disambiguating epidemiological criteria, it allows computational systems to precisely interpret case definitions, facilitating automated classification and seamless integration into modern digital surveillance pipelines.

In conjunction with the OSD format, this work presents the inaugural dataset of its kind: a collection of machine-readable case definitions for a plethora of diseases from a network of countries spanning 5 continents: the Americas, Europe, Africa, Oceania, and Asia. True interoperability between jurisdictions, reproducible research, and a foundation for more responsive, data-driven approaches to disease surveillance and public health emergency management are all enabled by this common “language.”

Case definitions play a critical role in public health and epidemiological research. However, they often suffer from ambiguity and a lack of standardization. This limits their utility for computational processing and interoperability. This study addresses the critical gap in translation between human-readable and machine-processable case definitions. To accomplish this, the study has three main goals: (1) to create the OSD, a standardized, machine-readable format that preserves the logical complexity of case definitions while eliminating ambiguity, (2) to enable the conversion of the machine-readable format to free text and vice versa, which is a necessary operational feature, and (3) to compile the first extensive dataset of structured case definitions that covers various diseases and jurisdictions. This study aims to support future advances in public health surveillance, syndromic surveillance, and broader epidemiological research by pursuing these goals.

## Methods

### Overview

This section describes the methodology used to develop the OSD format, dataset, and supporting tools. We took an iterative approach to the development process. The format evolved through continuous refinement as we collected case definitions, which became our dataset, and created supporting tools. Although this section is organized into distinct subsections for clarity, these components were developed concurrently and informed each other throughout the research process.

### Definitions Dataset

Case definitions have specific guidelines regarding writing style. These guidelines emphasize simplicity and conciseness and encourage the use of a narrative format [1]. Many governmental [24-26] and public health organizations [6,27] publicly share their case definitions to ensure that health workers and the public can access information about monitored diseases and their characteristics.

To identify scientific papers mentioning datasets related to case or syndrome definitions, we searched for the keywords *case definition AND dataset* and *syndrome definition AND dataset* across various scientific sources, as shown in Table 1. The choice of terms reflects the core concepts of interest, case definitions, and their association with publicly available or structured datasets. Our goal was to cast a broad net without overly constraining the results, so we used simple, inclusive phrases and applied no filters based on time or language. However, despite these efforts, we were unable to identify any relevant datasets.

Table 1 summarizes the results of searches conducted in 2 scientific article databases (PubMed and OpenAlex) and 3 dataset platforms: Kaggle, Hugging Face, and Harvard Dataverse. The searches yielded 286 results for “case definition dataset” and 12 results for “syndrome definition dataset,” but none of these were actual datasets for case or syndrome definitions.

**Table 1.** Search queries for case and syndrome definition datasets accessed on March 24, 2025.

Source	“case definition” AND “dataset”	“syndrome definition” AND “dataset”
PubMed [28]	53 results	0 results
OpenAlex [29]	213 results	12 results
Kaggle [30]	4 results	0 results
HuggingFace [31]	2 results	0 results
Harvard Dataverse [32]	14 results	0 results
Total	286 results	12 results
Confirmed datasets	0	0

Because our initial search yielded no results, we took a targeted approach to compile case and syndrome definitions for our dataset. Our methodology was a multistep heuristic process.

First, we focused exclusively on World Health Organization (WHO) member countries. Second, to ensure balanced representation, we aimed to include countries from each of the following continents: the Americas, Europe, Africa, Oceania, and Asia. Third, for each country, we began with the search query <country name> AND case definitions; if no relevant results were found, we successively tried *<*country name> AND syndromic surveillance definitions and <country name> AND syndrome definitions. Fourth, we aimed to include at least one definition from at least 10% of the countries within each continent. Once this target was met, we moved on to the next country and then the next continent. When a country provided multiple definitions on a single webpage or PDF, we selected a disease or group of diseases accordingly. DuckDuckGo was our primary search engine. For every successful find, we exported the web page or PDF file featuring case definitions. Since a page or file may contain multiple definitions, we extracted the text to create a machine-readable version. Among WHO member countries, Japan, Indonesia, Russia, and Cuba had no public definitions.

Ultimately, we collected a total of 40 case definitions. This collection comprises 36 national and regional definitions that collectively represent 60 countries. This coverage is reached primarily because a single regional definition from the Pacific Public Health Surveillance Network accounts for a group of 22 nations. Of the remaining 4 definitions, 3 are from continental organizations (the Pan American Health Organization, the European Centre for Disease Prevention and Control, and the Africa Centers for Disease Control and Prevention) and 1 is from the WHO as a global entity.

As we gathered definitions, common themes surfaced across various diseases and regions, including symptoms, diseases, epidemiological links, laboratory tests, and medical evaluations. We also recognized recurring logical patterns in how definitions combined criteria. Operators like AND, OR, and especially AT LEAST were frequently used. The AT LEAST operator called for a specific number of criteria to be met, as illustrated in definitions such as “fever AND at least two symptoms from: cough, headache, loss of smell, back pain.” Through careful analysis of existing definitions and relevant scientific literature [1,4,13], we pinpointed important metadata elements that, while often found in the broader context of portals and publications, were not consistently included with the definitions themselves. These insights led to the continuous development of our OSD format, which we cover in detail in the Schema subsection.

The dataset is organized into sections for human and machine readability. The human-readable section contains original PDF publications, which may cover multiple diseases, as well as TXT files that contain case definitions converted into our format. To ensure consistency, all web-published definitions were exported to PDF. The machine-readable section includes JSON representations of these definitions that have been validated with the Ajv validator [33]. The dataset is available on the Open Syndrome Initiative (OSI) page at HuggingFace and GitHub.

### Schema

**Figure 2.**
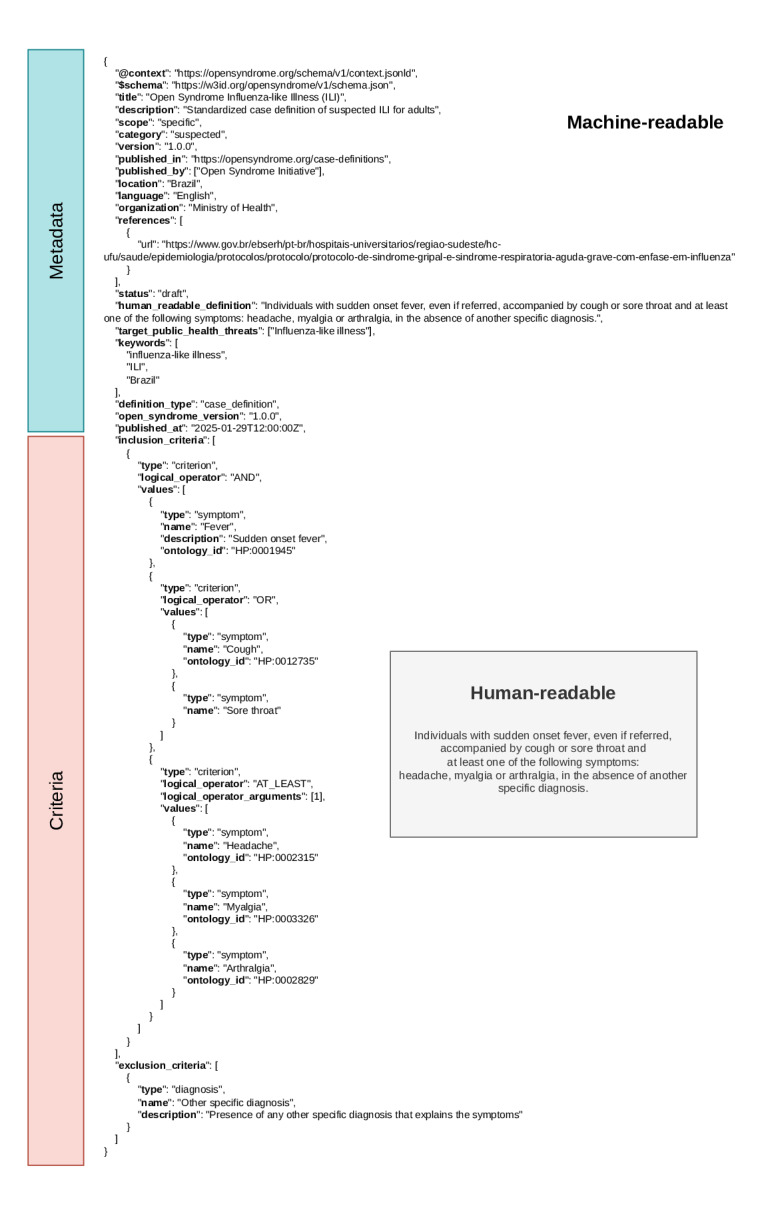
The human- and machine-readable case definitions of influenza-like illness from the Ministry of Health of Brazil are in the Open Syndrome definition format. The human-readable case definition is shown in the gray box on the right. The machine-readable definition is on the left and uses the JSON format proposed by this work. There are two additional boxes on the sides: the green upper box indicates the metadata fields, and the pink box outlines the inclusion and exclusion criteria.

The OSD format converts traditional, narrative case definitions into a structured JSON schema with defined properties and types. These properties were derived through an in-depth review of a wide range of existing case definitions to ensure they reflect common structural and semantic elements found in real-world usage. When analyzing case definitions of different public health threats from various countries and continents, we identified consistent patterns in information groups. More details are provided in the Definitions Dataset subsection. We observed that the majority (217/407, 53%) relied on symptoms to describe conditions, followed by diagnosis (34/407, 8%). Additionally, half of the case definitions included criteria that resembled logical operators (such as AND, OR, and AT LEAST) to group-related conditions. Based on these observations, we developed a nested structure using a data-driven approach combined with our established principles. As illustrated in Figure 2, we organized the format into 2 main groups of information: metadata and criteria. We describe both groups of information below.

First, the metadata information group provides essential context about the definition itself. This includes version information, scope (broad or sensitive vs narrow or specific), publication details, the responsible organization, the language used, and other provenance information. Typically, in narrative formats, this metadata is presented implicitly within published documents or websites (eg, European Centre for Disease Prevention and Control case definitions [6], WHO Outbreak Toolkit [34]). We enabled efficient information retrieval, version tracking, and proper attribution by explicitly structuring this information in a machine-readable format. Furthermore, the metadata information group consists of metadata properties that capture essential contextual information about the case definition, ranging from basic identification (title and description) to publication details (published_in, published_at, and authors) and geographical context (location and language). We included properties for tracking the status and version of the case definition itself and the OSD schema. Most metadata elements are derived directly from the original case definitions, though several properties, such as Open Syndrome Version, $schema, and the JSON-LD @context, are specific to our format’s technical infrastructure and initiative, which we discuss further in the OSI section. These and other individual metadata properties and their meaning can be seen in Table 2.

Table 2 outlines the first-level properties of the Open Syndrome Definition format, dividing them into metadata and criteria. The metadata category contains information about the format and syndrome, including location, organization, and version. The criteria category contains structured information that defines the syndrome itself.

**Table 2.** Breakdown of the format by group of information types.

Key components and their properties	Description
Metadata	
$schema	URI^a^ pointing to the JSON^b^ schema that validates this document
@context	JSON-LD^b^^c^ context mapping for semantic interoperability
@type	Semantic class (eg, OSD^d^: case definition or OSD^d^: syndrome definition)
ID	Unique ID from the OSI^e^
Title	Case definition title
Description	A detailed description of the definition
Human-readable definition	A human-readable description of the case definition for user interfaces
Scope	Level of specificity. Options: broad or specific
Created at	Date when the definition was created
Published in	Source or platform where the definition was published
Published at	Date and time in UTC^f^ when the definition was published
Published by	List of case definition publishers in the OSI^e^
Authors	List of the case definition authors
Location	Geographical location relevant to the schema’s application
Language	Language in which the definition is written (eg, English and Spanish)
Organization	Organization or initiative responsible for the schema maintenance
Status	Current status of the definition. Options: draft, published, and deprecated
Keywords	Keywords related to a definition (eg, COVID-19 and mpox, outbreaks)
Target public health threats	List of public health threats that this definition targets
Notes	Any notes that may be relevant to this definition
Category	Case definition categories [1]. Options: confirmed, probable, suspected
Version	Case definition version to be established by the author
Open Syndrome version	Open Syndrome Definition schema version. Currently: v1 (version 1)
Definition type	Case definition or syndromic indicator
Surveillance system type	Type of surveillance system this definition is
Criterion	
Criterion (meta-type)	Set of properties and operators to select a case (details in Table 3)
Inclusion criteria (criterion)	Criterion used to include a case
Exclusion criteria (criterion)	Criterion used to exclude a case
References	Scientific references that have supported this definition

a URI: uniform resource identifier.

b JSON: JavaScript object notation.

c LD: linked data.

d OSD: Open Syndrome definition.

e OSI: Open Syndrome initiative.

f UTC: Coordinated Universal Time

Second, the inclusion and exclusion criteria specify the conditions that determine whether a case meets the definition or should be excluded. These conditions, referred to as criteria, can be combined using logical operators (AND, OR, and AT_LEAST) to express complex clinical relationships. We organized the data into key and value pairs (properties) within the JSON schema, enabling computational systems to interpret simple and compound conditions precisely. This structured approach eliminated ambiguities often present in narrative text [35] while preserving the clinical intent of the original definitions. The criteria properties form the backbone of the format and are structured to capture the logical relationships inherent in case definitions. The format distinguishes between inclusion and exclusion criteria, both built upon our criterion meta-type, as described in Table 3. This structure enables the representation of complex clinical reasoning within a machine-readable framework.

**Table 3.** Criterion meta-type properties (summary of properties of a criterion and a fundamental component of a definition).

Property	Description	Usage details
Type	Type of criterion. Options: criterion, syndrome, symptom, diagnosis, diagnostic_test, professional_judgment, epidemiological_history, and demographic_criteria	The type property is mandatory, followed by name or values
Name	Criterion label	—^a^
Description	Detailed description of the criterion	—
Ontology ID	The compacted identifier from an ontology (eg, Disease Ontology (DOID))	Examples: hpo:0002045 and mondo:0020674. Facilitates semantic interoperability
Logical operator	Keywords that represent a logical operation on criteria. Options: AND, OR, and AT_LEAST	The logical operator AT_LEAST must be used with the number specified in logical_operator_arguments
Logical operator arguments	List of arguments to be passed to the logical operator	—
Attribute	The referred attribute, for example, body temperature, age, and onset	It is used in composition with operator and value
Value	The reference value for the referred attribute. It could be of any data type because it can represent anything in the real world	Examples: true, 37.6, abnormal but non-specific bowel gas pattern. This property is used in composition with attribute and operator
Operator	Comparison and matching operators. Options: >, >=, <, <=, ==, !=, regex	It is used in composition with value and attribute
Regex pattern	Regular expression for evaluation and pattern matching	It is used in composition with operator
Regex flags	Regular expression flags for extra configuration	It is used in composition with regex_pattern
Code	A system-agnostic diagnosis code object that holds system, code, and display	Useful to represent values from the *ICD*^b^, SNOMED CT^c^, and others
Values	A list whose types are a criterion	The criteria items should be unique

a Not applicable.

b *ICD*: *International Statistical Classification of Diseases and Related Health Problems*.

c SNOMED-CT: Systematized Nomenclature of Medicine—Clinical Terms.

To ensure true semantic interoperability, the OSD format is designed as a valid JSON-LD document. JSON-LD allows a standard JSON file to act as a graph of linked data simply by defining a context. Each definition includes a @context field that explicitly maps the schema properties to universally accepted medical ontologies and vocabularies, such as Schema.org [36], BioSchemas [37], and the Human Phenotype Ontology (HPO) [38]. This transformation gives explicit semantic meaning to each string field, allowing automated systems to interpret the exact biological or epidemiological concept behind the text [39]. Furthermore, to guarantee long-term machine readability, every generated JSON file includes a $schema property pointing to a persistent, community-governed uniform resource identifier hosted via the W3C permanent identifier infrastructure (eg, [40]). This approach provides a stable reference to the validation schema regardless of future repository migrations.

To capture the logical nature of the collected definitions, we implemented a flexible attribute-operator-value pattern. This pattern can accommodate diverse clinical observations ranging from simple Boolean conditions to complex pattern matching through regular expressions. The format supports numerical comparisons (>, >=, <, <=, ==, and !=) and text pattern matching (eg, regular expressions and through regex), enabling precise representation of quantitative thresholds and textual patterns. Please note that logical operators are available at the criteria level (see the Schema section). To handle semantic mapping, the format provides two specialized properties: ontology_id is used to represent compacted uniform resource identifiers for broad medical concepts (eg, HPO or MONDO Disease Ontology [MONDO] identifiers), while the code object is used to capture specific, system-agnostic clinical billing or diagnostic codes from systems like *ICD* (*International Statistical Classification of Diseases and Related Health Problems*) [41] or SNOMED CT (Systematized Nomenclature of Medicine—Clinical Terms) [42].

The criterion structure can be defined recursively. The values property enables criteria to be nested within other criteria. This design allows us to represent complex logical relationships, ranging from simple symptom lists to intricate decision trees with multiple levels of criteria. With this property structure, we strike a balance between human readability and machine interpretability. We preserve the clinical logic of narrative case definitions while enabling computational processing and analysis.

### OSD Format

Our schema was developed iteratively, resulting in the creation of the OSD format. We designed the format to include both inclusion and exclusion criteria, reflecting common patterns found in various case definitions. We extracted essential metadata components, such as location, publication date, title, authors, and citation details, from the publishing websites and accompanying papers. This framework for metadata ensures proper attribution and provides contextual information for each definition.

We manually adapted the case definition texts into JSON format and used validation tools to verify their structural integrity and compliance. Transforming the texts from human-readable to machine-readable format revealed additional patterns and edge cases, which informed iterative refinements to the format. Throughout this iterative development process, we prioritized flexibility while maintaining structural consistency. This allowed the format to represent definitions across varied health systems, geographical regions, and clinical contexts.

### Tooling

As we developed the OSD format and dataset, we realized that supporting tools were necessary to facilitate the conversion of human-readable case definitions into machine-readable formats. We developed a Python-based toolkit to streamline this process, enabling researchers and public health professionals to efficiently translate traditional text-based case definitions into structured JSON representations, and vice versa. The toolkit used Python version 3.11 (Python Software Foundation).

We implemented a conversion utility that leverages large language models (LLMs) to automate the structuring process. To accommodate different user preferences and computational resources, the tool supports local, privacy-preserving deployment via Ollama [43] (version 0.9.6), as well as direct application programming interface (API) integration with major cloud providers, including OpenAI, Anthropic, Google Gemini, Mistral, and DeepSeek. We systematically evaluated multiple local models, including llama-3.2 (Meta) [44], mistral-7b (Mistral AI) [45], and deepseek-r1 (DeepSeek; both 7b and 8b variants) [46], medllama2 (Siraj Raval) [47], and qwen2.5-coder (Alibaba Cloud) [48]. We evaluated the performance of the models based on their ability to comprehend medical terminology accurately and remain faithful to the original clinical meaning. Of the models evaluated, llama-3.2, mistral-7b, and deepseek-r1 demonstrated adequate performance for the conversion task. Mistral-7b was selected as the recommended default due to its optimal balance of accuracy and resource requirements.

Additionally, we developed a reverse conversion function that transformed machine-readable JSON syndrome definitions into human-readable formats that support multiple languages. This bidirectional conversion capability ensures accessibility for diverse user groups and facilitates international collaboration in syndromic surveillance systems.

To evaluate the structural and semantic interoperability of the OSD format, we developed a data filtering prototype designed to process tabular health datasets against our standardized JSON-LD schema [49].

### OSI

To promote the adoption and ongoing development of the OSD format, we created the OSI, a collaborative community platform. The OSI serves as the central hub for all OSD-related resources and provides an infrastructure for sharing, collaboration, and knowledge exchange. We cover the methodology behind the website and its functionalities in the following text.

First, to ensure transparency and promote collaboration, we developed the OSI website using open-source tools and libraries. We use Hugo [50] (version v0.145.0) to create the static website, which is built using Markdown [51] files. This allows other community members to easily improve or expand the website content with new blog posts or tutorials.

**Figure 3.**
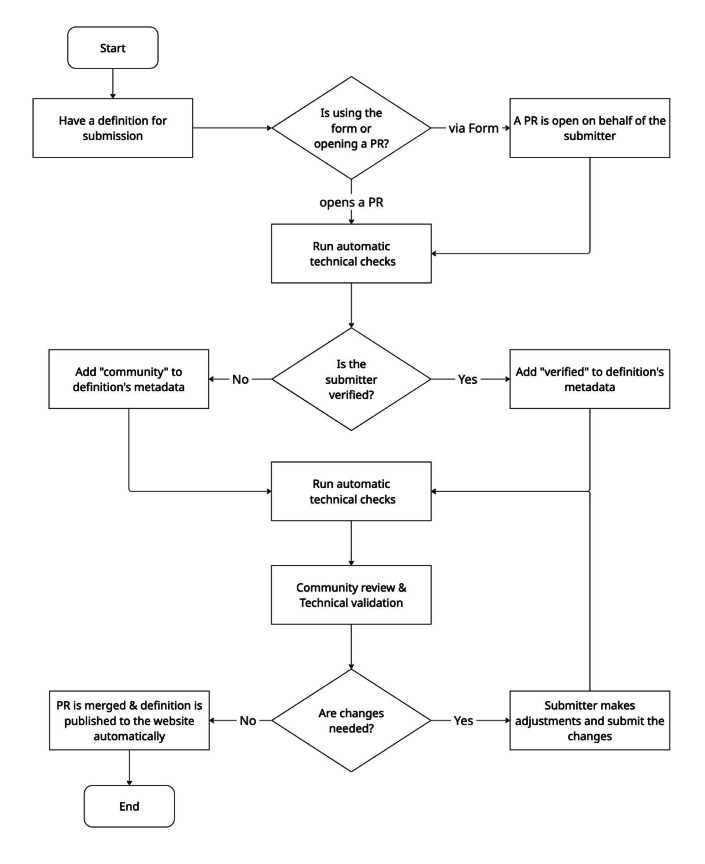
Open Syndrome Initiative contribution workflow. PR: pull request.

Second, the website provides a user-friendly contribution workflow for submitting new definitions. This functionality is enabled through pull requests or a simplified web interface, as depicted in Figure 3.

All contributions undergo a community review and are published on our website after receiving approval and technical validation. When a definition is submitted via the form, one of the maintainers will create a GitHub pull request on the user’s behalf in our repository. Despite their technical skill level, the user can view the submission publicly available in our repository and provide feedback. This process helps us maintain a clear history of contributions and ensures that all definitions are version-controlled. The submission then follows the same process as any other pull request, including validation and community review. We developed the entire toolkit as open-source software and hosted it on GitHub to enable collaborative development and continuous improvement. To ensure quality and authenticity, we verify the institutional affiliations of first-time contributors.

We implemented the automated validation workflows using GitHub Actions to maintain quality and consistency. These workflows verify JSON schema compliance for each definition and validate new submissions against established formatting requirements. The workflow verifies the conformity of submitted JSON files with a predefined schema. This process ensures that the JSON structure is well-formed, that all required fields are present and correctly named according to the schema specifications, and that the values adhere to the expected data formats (eg, URLs and dates). We open-sourced the entire toolkit and hosted it on GitHub to enable community contributions and continuous improvement through collaborative development. Our workflow is straightforward: we use a GitHub repository as the central hub where all definitions are stored and managed. Anyone can submit a new definition through the submission contact form or directly via a GitHub pull request. Additionally, individuals can verify their affiliations and become recognized contributors. To do so, they must provide a valid email address and their organization’s name through the verification form. Definitions submitted by verified contributors receive a verified tag.

Third, the website features an interactive graph visualization of the definitions dataset that we developed using D3 [52] (version v7.9.0), an open-source JavaScript library for visualizing data, and D3 force-directed graph layout using velocity Verlet integration. This feature allows users to explore the dataset intuitively by panning, zooming, and viewing tooltips that display important information about each criterion. The visualization automatically updates to reflect changes in the underlying dataset, so users always see the most current information.

Fourth and last, the web interface includes extra documentation with instructions and suggestions on how to read, work with, and upload case definitions. For those unfamiliar with the format, we provide extensive instructions on how to convert text-based definitions to the OSI format using our tooling.

### Ethical Considerations

This study was exempt from institutional ethics review as it exclusively uses publicly available case definitions published by governmental and public health organizations. No patient data, personal information, or human subjects were involved.

## Results

### Overview

This section examines the structure and characteristics of the case definition format. It introduces the first case definition dataset and its maintenance tools. Finally, it presents the OSI, a collaborative community built around this ecosystem.

### The OSD Format

The OSD format was designed to accurately preserve the clinical meaning of case definitions in a machine-readable representation. Examining various case definitions reveals that, despite differences in language and style, they consistently strive to be clear, simple, and concise [1,53]. We have also deliberately incorporated these essential qualities into our format.

The OSD format is a JSON schema, a standard for defining the structure and rules of JSON data. With a JSON schema, one can describe the overall structure of a definition, including its inclusion and exclusion criteria, metadata, properties, and more. The OSD format transforms traditional narrative case definitions into structured, machine-readable representations. The version used for the proposed OSD format is draft 2020‐12 [54]. For example, a free-text definition for influenza-like illness (right side of Figure 2) becomes a structured JSON object (left side of Figure 2) specifying each criterion with precise logical relationships and the metadata of this definition.

This structured format explicitly defines signs, quantitative attributes, and temporal constraints, which could be ambiguous in a free-text narrative. Additionally, it incorporates metadata, such as provenance and version history, which is often missing from traditional formats. This structured approach enables computational systems to accurately interpret case definitions, thereby facilitating automated case classification, cross-jurisdictional comparison, and application in ML pipelines. Several core principles guided our design decisions.

1. Preservation of clinical meaning: Maintaining the integrity of case definitions while keeping a structured format and preserving essential clinical details.
2. AI Readiness: Designing with computational processing in mind to enable the large-scale analysis and application of AI.
3. Interoperability: Ensuring seamless integration with existing and future systems across different platforms, following the Findability, Accessibility, Interoperability, and Reusability (FAIR) principles [55] and promoting interoperability [56].
4. Openness and Accessibility: Developing a reusable, open format that is free to use and not tied to proprietary platforms.
5. Decentralization: Allowing for independent implementation across websites, scientific publications, and surveillance systems without central control.
6. Versionability: Supporting the evolution of definitions over time with clear tracking of changes.

The format addresses several critical challenges in the current landscape. These challenges include a lack of standardization in case definitions, potential ambiguity in text-based definitions, fragmented information caused by inconsistent metadata, and barriers to large-scale AI applications resulting from format inconsistencies. Even though the technical nature of the format might require some knowledge of JSON, we alleviate this by providing user-friendly tools described in the Tooling section.

This format has several promising applications, including testing definitions against electronic health record data, creating ML models for automated case detection, comparing disease definitions across countries, and enhancing reproducibility in epidemiological research.

While our collection of data effectively documented a wide array of diseases and methods of definition, we recognize the necessity for subsequent improvements. To that end, we included a version field in the format specification. This allows us to maintain backward compatibility and accommodate future improvements as the field evolves. This versioning approach allows the OSD format to adapt to emerging needs while preserving access to historical definitions. The resulting format balances machine readability with a faithful representation of the original clinical intent. This enables automated processing while preserving the essential diagnostic criteria established by public health authorities.

### Definitions Dataset

The first comprehensive dataset of machine-readable case definitions was developed, including definitions from 60 countries, 3 continental organizations, and one global organization. The dataset contains 40 case definitions for various diseases and is available in 3 formats: OSD JSON, plain text, and the original PDF publications. The definitions cover a wide range of disease categories: vector-borne illnesses (n=8), viral respiratory diseases (n=7), bacterial diseases, vaccine-preventable diseases (n=6 each), gastrointestinal diseases (n=5), hemorrhagic fevers (n=4), nervous system and other infectious diseases (n=2 each), and climate-related and noninfectious conditions (n=1 each).

**Figure 4.**
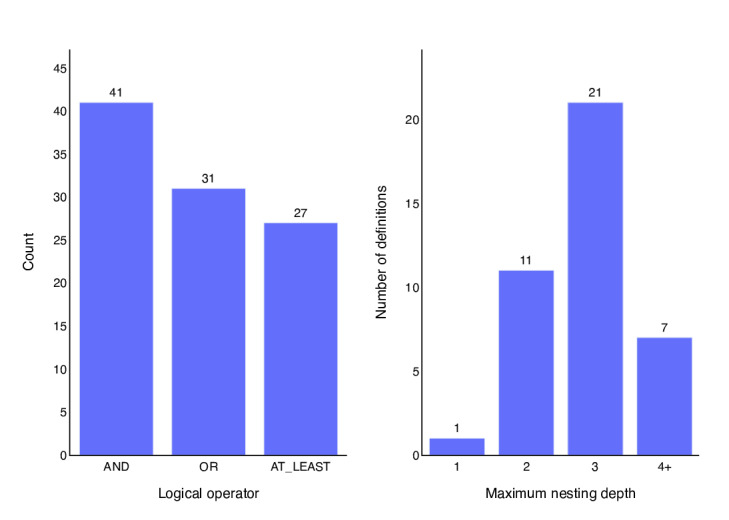
Distribution of maximum nesting depth and the frequency of logical operators in the machine-readable dataset definitions.

To quantify the structural characteristics of the collected data, we assessed the logical complexity of the definitions within our dataset (Figure 4). Regarding logical operator usage, AND was the most frequent operator (n=44), followed by OR (n=33) and AT_LEAST (n=28). Regarding maximum nesting depth, most definitions had a depth of 3 (n=21), followed by depth 2 (n=11), depth 4 or more (n=7), and depth 1 (n=1). The analysis revealed that the majority of the definitions use up to three levels of nesting depth to represent their clinical criteria.

Measles is the most represented disease, with 4 definitions. Cholera, influenza-like illness, and COVID-19 each have two definitions. This likely reflects their status as priority conditions in national and international public health surveillance initiatives, in which consistent case definitions are crucial for monitoring morbidity and responding to outbreaks. The remaining 30 diseases, ranging from common conditions like dengue fever to rare diseases like Lujo hemorrhagic fever, are each represented by one definition.

**Figure 5.**
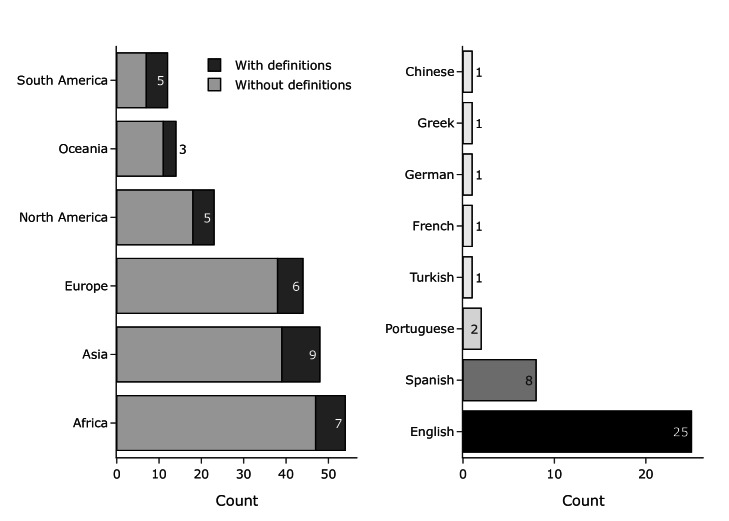
Distribution of definitions by location and language.

Additionally, we ensured geographic diversity by selecting at least 10% of countries from each continent, based on the number of countries represented in the United Nations, to guarantee diversity in geographic representation. For example, the Pacific Islands are represented collectively through the PPHSN. This collaborative effort involves 22 Pacific Island countries and territories that develop case definitions together. Although using English for our search terms may have introduced some bias, the definitions were available in a range of languages, as depicted in Figure 5.

### Data Filtering Prototype

**Figure 6.**
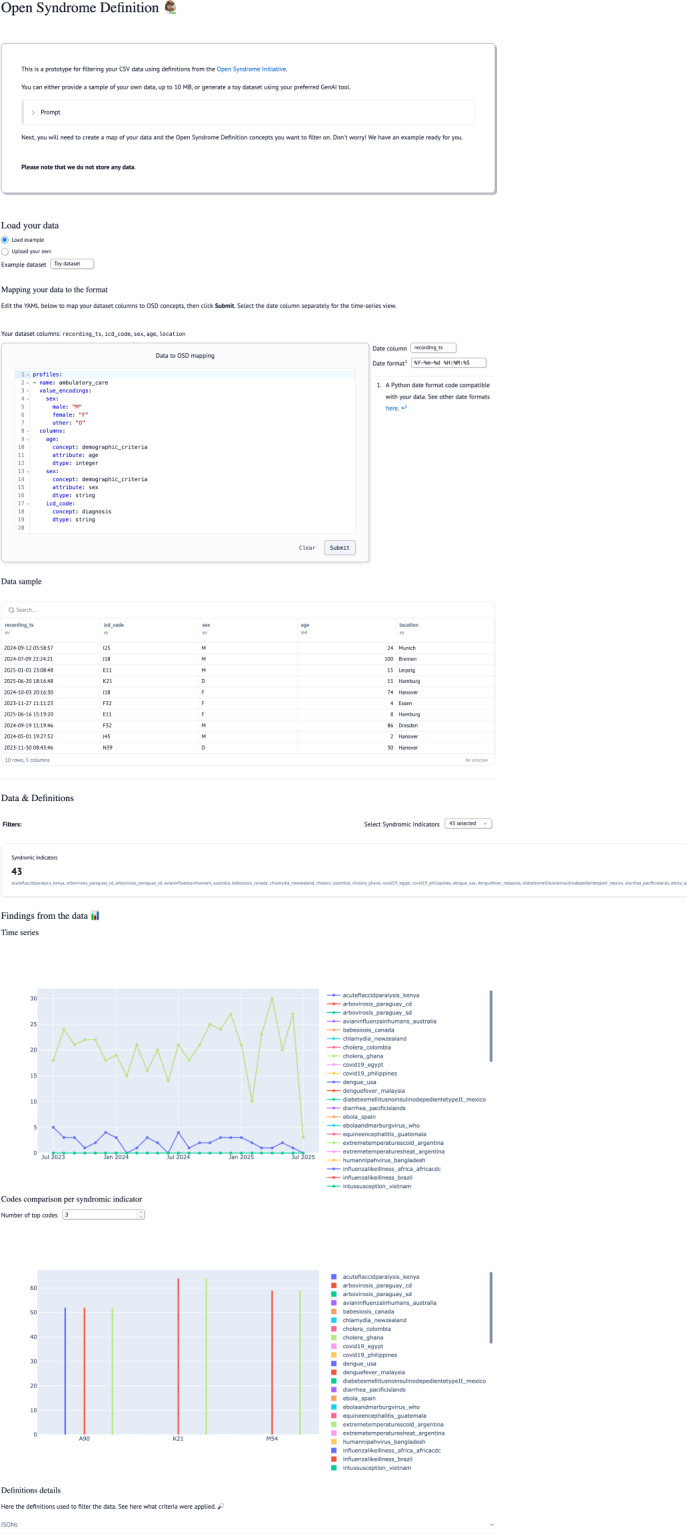
The data filtering prototype allows users to upload a sample of their own data or interact with a toy dataset to see which cases are matched with Open Syndrome definitions available on the website.

To demonstrate the practical utility and downstream applicability, we successfully implemented a data filtering prototype as a proof-of-concept. This web-based tool, publicly accessible at the Open Syndrome Initiative web page [57] (see Figure 6), enables users to upload their own tabular health datasets and apply our standardized case definitions to automatically identify matching patient records.

By leveraging the newly integrated JSON-LD schema, which maps definition criteria to established medical ontologies (eg, HPO and Disease Ontology), the prototype ensures semantic consistency even when processing data from diverse health systems.

This successful implementation validates the structural and semantic interoperability of our format, showcasing its capacity to facilitate automated, cross-platform epidemiological surveillance without ambiguity.

### Tooling

To overcome barriers to adopting our machine-readable case definition format, we developed a Python command-line tool leveraging LLMs via Ollama to convert between traditional text definitions and our structured format [43]. The library offers two main features: it automatically generates structured JSON representations from case definitions and converts JSON definitions back to human-readable text for verification and sharing.

The tool reliably converts between human- and machine-readable formats using either locally hosted LLMs (via Ollama) or prominent cloud-based models. We do not provide a quantitative evaluation of the tool, as this lies beyond the scope of the current work and reflects its auxiliary role in our study. However, our qualitative analysis indicates that Mistral [45] yields the most reliable results. The tool demonstrates strong fidelity, avoiding hallucinations and accurately translating text into a machine-readable format. This qualitative assessment was conducted by two coauthors (APGF and AA). The library was also designed to be flexible, offering two distinct customization options for the conversion process.

First, users can select their preferred LLM model for conversion. Second, users can specify their preferred output language, enabling multilingual text generation, which is essential for the international adoption of public health systems. By incorporating this flexibility into our library, we ensure local usability and minimize dependencies.

We made the tool available through the Python Package Index under the namespace *opensyndrome* and the project’s GitHub repository, enabling integration into existing workflows. We aimed to bridge the gap between technical and nontechnical users by providing extensive documentation on the format, library, and database. This makes the OSD format and its accompanying tools and data accessible to health workers in organizations without dedicated data teams.

### OSI

The OSI is a central platform for sharing and accessing case definitions. The initiative’s ecosystem facilitates the practical application of standardized case definitions in public health surveillance. It does so by providing a website where users can search, browse, and analyze definitions. The website displays and indexes definitions from the OSI GitHub repository, offering the most current versions and statistical information about the definitions. Users can contribute definitions or suggest modifications via GitHub pull requests or an online form. This dual approach ensures participation from technical and nontechnical users alike. To ensure high-quality dataset definitions, we implemented a two-stage validation process that combines automated static checks with manual semantic verification upon uploading new definitions. The first stage detects missing required fields, formatting inconsistencies, and invalid property types and provides immediate feedback for correction. The second stage involves manually reviewing each definition following the automated checks to semantically validate the uploaded data and ensure its accuracy. The OSI also provides supplementary resources, such as documentation and educational blog posts on best practices.

These resources [58-60], alongside the technical infrastructure, support a community of practice around standardized syndrome definitions and enhance global health surveillance capabilities.

## Discussion

### Principal Findings

This work introduces the OSD format, a novel approach to representing case definitions in machine-readable form. Alongside this format, we presented the first comprehensive dataset of case definitions and developed supporting tools through the OSI to foster a community of practitioners. These contributions address a significant gap in public health surveillance infrastructure, as standardized digital representations of case definitions have been notably absent.

Our findings demonstrate that the OSD format is flexible enough to accommodate case and syndrome definitions in various clinical contexts. The format’s nested structure effectively captures the logical relationships present in traditional case definitions. Based on our curated dataset of structured definitions, we found that 53% (217/407) of the definitions primarily rely on symptom-based criteria. We assessed the logical complexity of each definition by analyzing the depth of nested Boolean expressions and observed that, although logical groupings were common, nesting rarely exceeded three levels. This suggests that complex clinical conditions can be represented within relatively constrained logical structures.

The development process revealed important insights about case definition structures internationally. The predominance of symptom-based criteria across geographical regions and disease categories indicates a universal approach to case definitions, despite variations in health care systems and resources. Additionally, the recurring use of logical operators across definitions from different origins indicates a natural convergence toward structured diagnostic thinking, which our format explicitly codifies.

Despite these promising results, our work has several limitations that warrant consideration. First, while the current dataset is diverse, it is relatively small, containing only 40 case definitions. Although our selective sampling technique covered all continents and major health care systems, the limited sample size may not capture the full range of definitions used globally. A further limitation of the current study lies in the evaluation of semantic fidelity during the conversion process. At this stage, the high semantic fidelity of the machine-readable definitions was assessed qualitatively through manual review by two domain experts. While this human-in-the-loop approach established a highly accurate baseline and ensured that clinical intent was preserved, it is inherently resource-intensive and difficult to scale. Furthermore, the lack of established quantitative benchmarks or baselines in this specific field makes comparative evaluation difficult.

We recognize that this initial, one-of-a-kind dataset will serve as a solid foundation for work ultimately developed by and for the community. This is why we developed additional tools to ensure a streamlined, user-friendly expansion process accessible to nonexperts. Additionally, the multilingual nature of our source definitions enriches the dataset’s diversity but introduces complexity in accurately translating clinical concepts across language barriers. To address this issue, we have included a versioning field in the format of our open-source repositories. This enables the community to propose modifications or corrections for any errors or omissions. Furthermore, the lack of established benchmarks or baselines in this field makes comparative evaluation difficult.

Nevertheless, our work establishes the OSD format at the intersection of significant developments in public health informatics. Its JSON-based structure allows for seamless integration with AI and ML projects, enabling researchers to use these standardized definitions when developing and validating algorithms. As Wang et al [61] noted in their work on automating case identification, structured case definitions can significantly improve the efficiency of surveillance systems. Similar applications have demonstrated that standardized case definitions enhance the assessment of information while alleviating the burden on physicians and clinic managers [62-65].

Our approach’s interoperability means that any software capable of parsing JSON can work with these definitions, significantly lowering technical barriers to adoption. This universal accessibility promotes international collaboration and knowledge sharing, enabling public health officials to quickly adopt and implement definitions from other regions. This capability could substantially accelerate the establishment of effective surveillance systems during disease outbreaks, especially in settings with limited resources. While established health care interoperability standards, such as Fast Healthcare Interoperability Resources and Observational Medical Outcomes Partnership Common Data Model, provide comprehensive frameworks for structuring broad medical information, they often require substantial infrastructural investment, specialized technical expertise, and complex data mapping. These requirements can present significant barriers to entry, particularly for resource-constrained public health departments or during rapid, localized outbreak responses. In contrast, the OSD format is purposefully designed to be lightweight and accessible. By using a straightforward JSON-LD schema focused exclusively on epidemiological case definitions, it allows health workers and researchers to standardize their criteria immediately without needing to overhaul their existing IT systems. Table 4 provides a structured comparison across key dimensions. The OSD format is not intended to replace these robust electronic health record standards; rather, it serves as an agile, domain-specific complement. It dramatically lowers the technical barrier to entry for epidemiologists while retaining the structural capacity to be mapped to Fast Healthcare Interoperability Resources (eg, Measure or PlanDefinition) for downstream institutional integration.

By grounding the OSD format in semantic web principles, our work directly connects to and builds upon large-scale existing efforts aimed at making medical diagnoses machine-readable. Initiatives such as the representation of rare diseases in BioSchemas and the MedicalCondition and MedicalCause classes in Schema.org have demonstrated the vast potential of linked data in health care. The OSD format complements these efforts by providing a specialized, deeply nested structural representation specifically tailored for public health surveillance and early warning systems. Future work will explore formally suggesting an epidemiological “MedicalCase” or “Criterion” class extension to the BioSchemas community, thereby contributing our public health modeling back to the broader semantic web ecosystem.

Looking ahead, the OSD format offers several promising avenues for future development. Users will be able to download definitions from various global sources and estimate the number of potential cases they could identify in their local data. This feature will speed up the development and adoption of locally optimized definitions and facilitate the implementation of ML models that leverage this standardized format. As more institutions contribute to and adopt this approach, we anticipate accelerated collaboration across borders and more responsive surveillance capabilities worldwide.

While our present efforts are concentrated on case definitions, there is also considerable potential to broaden the ready implementation of our methodology to syndromic surveillance. The principle of syndromic surveillance is to monitor patients presenting with symptoms, chief complaints, or other nonlaboratory or confirmatory diagnoses. Public health experts coordinating syndromic surveillance map individual clinical codes to more generic syndromic indicators. While these are not case definitions, as they are purely data- and code-based, similar problems exist with the consistency of syndromic indicators internationally.

Using OSD to present the code mappings that underpin syndromic indicators and to standardize these indicators internationally would support the global effort to coordinate syndromic surveillance between countries.

The versioning system in our format makes it easy to adapt to changing needs while also ensuring that older versions remain compatible. This addresses a crucial requirement for sustainable public health infrastructure. As the OSI expands its community of practitioners, we anticipate that the collective expertise will further refine the format and available definitions, thereby creating a robust ecosystem of standardized syndromic surveillance resources accessible to all.

In conclusion, the OSD format represents a significant step toward standardizing and digitizing case definitions for public health surveillance. It establishes a foundation for enhanced interoperability, collaboration, and automation in syndromic surveillance. The format’s potential applications extend beyond traditional public health contexts into clinical research, healthcare delivery, and emerging disease response.

**Table 4.** Comparison of Open Syndrome definition, CQL^a^ or FHIR^b^, and OHDSI^c^ Phenotype Library across key dimensions. CQL and the OHDSI Phenotype Library are mature, platform-coupled standards optimized for clinical care and retrospective research, respectively. The Open Syndrome definition distinguishes itself through minimal infrastructure requirements, a decoupled definition-execution architecture, and native support for cross-country interoperability and AI pipelines.

Dimension	CQL or FHIR	OHDSI phenotype library	Open Syndrome definition
Required infrastructure	FHIR-compatible EHR^d^ with CQL engine	OMOP^e^ CDM^f^ with ATLAS	JSON^g^ parser; optional Python package (opensyndrome)
Format and execution	Domain-specific language; coupled to CQL engine	Platform-generated JSON; executed via HADES^h^ (R)	JSON-LD^i^; decoupled from execution pipeline
Ontology support	SNOMED^j^, LOINC^k^, RxNorm via FHIR bindings	OMOP Vocabulary (Athena); locked to CDM	Agnostic; reuses HPO^l^, MONDO^m^, *ICD*^n^, SNOMED via JSON-LD
Cross-country interoperability	Depends on FHIR adoption	Depends on CDM adoption	Primary design goal; explicit provenance and location metadata
Multilingual support	Partial	No	Yes
ML^o^ or AI pipeline integration	Indirect via structured output	Via R with HADES package	Structured for direct ingestion into ML pipelines

a CQL: Clinical Quality Language.

b FHIR: Fast Healthcare Interoperability Resources.

c OHDSI: Observational Health Data Sciences and Informatics.

d EHR: electronic health record.

e OMOP: observational medical outcomes partnership.

f CDM: Common Data Model

g JSON: JavaScript object notation.

h HADES: Health-Analytics Data to Evidence Suite.

i LD: linked data.

j SNOMED: Systematized Nomenclature of Medicine – Clinical Terms.

k LOINC: Logical Observation Identifiers Names and Codes.

l HPO: human phenotype ontology.

m MONDO: MONDO disease ontology.

n *ICD*: *International Statistical Classification of Diseases and Related Health Problems*.

o ML: machine learning.

### Future Work

While our current dataset provides a robust baseline for validating the OSD format, we recognize the potential for scaling the repository. A major technical challenge remains the heterogeneity of source PDFs, which makes fully automated bulk extraction prone to errors. To address this, future development will introduce specialized extraction skills within our tooling. These modules will use a 2-step pipeline to first identify multiple conditions within a single document and then structure them, keeping a human in the loop for quality assurance. To enhance the quality assessment process, we intend to adopt automated validation frameworks, such as leveraging advanced LLMs as evaluators (LLM-as-a-judge), to methodically and quantitatively evaluate semantic fidelity in conjunction with our community-driven review process. Furthermore, as an open-source initiative, the platform is now fully equipped to receive community contributions, allowing the dataset to grow organically and incorporate definitions discovered through advanced search methodologies.

To enhance the semantic precision of this extraction process, we plan to implement deterministic mapping to standardized clinical codes. While LLMs excel at generating the initial structural hierarchy, assigning precise ontological codes requires a more rigid approach. Our tooling architecture will be updated to integrate with biomedical ontology repositories, such as the National Center for Biomedical Ontology BioPortal API. Through this integration, terms extracted by the LLM can be automatically queried against standard vocabularies (eg, SNOMED CT or *ICD-10* [*International Statistical Classification of Diseases and Related Health Problems Tenth Revision*]) to retrieve the most accurate concept identifiers, which are then validated by human experts and populated into the format’s code fields.

Additionally, to maximize the utility of the OSI platform, we intend to develop an interactive comparative tool. As the repository grows, this feature will allow researchers and public health officials to visually and logically compare case definitions for the same disease across different jurisdictions. This will facilitate the identification of surveillance discrepancies and support the harmonization of global syndromic indicators.

Finally, a significant future development is improving the AI-driven natural language processing techniques that transform narrative definitions into OSD-format representations. The local LLMs of the current system, which are accessed through Ollama, could be supplemented with BioBERT [66] or ClinicalBERT [67]. Additionally, the Structured Prompt Interrogation and Recursive Extraction of Semantics (SPIRES) framework [68], which combines zero-shot LLM prompting with deterministic ontology grounding, could complement our current extraction pipeline by improving identifier precision for HPO and MONDO terms. These models are specifically trained on multilingual and multicultural clinical datasets, which could improve parsing precision and flexibility. Furthermore, AI anomaly detection techniques (eg, isolation forest and transformer-based systems) could be used to automate the validation and correction of definitions, thereby improving the continuous quality of the dataset. AI-federated approaches would also enable collaborative algorithm development across jurisdictions while ensuring data confidentiality, which is essential for international disease monitoring and surveillance. Public health–federated learning frameworks, such as Flower [69] and PySyft [70], could train and evaluate OSD format–based ML models across regions without sharing sensitive health information. This would improve cross-border analysis, equity, fairness, and public health. OSD format may advance from a defined structure for collaborative model development by progressing in this way.

### Conclusions

The OSD provides a flexible, interoperable, and machine-readable representation of case definitions. This innovation enables public health professionals, researchers, and technically proficient individuals to exchange epidemiological information more efficiently and consistently. It also enables the broader application of AI and machine learning techniques to public health data. Along with the format, this work introduces the first dataset of case definitions available in both human- and machine-readable formats. The dataset demonstrates the adaptability of the OSD format across various diseases and geographic settings, paving the way for global comparative analyses of case definition methodologies. To promote collaboration, we have launched the OSI, a platform where users can share definitions and access tools for converting between human- and machine-readable formats. Public health is inherently collaborative, and the OSD format contributes to this shared effort by promoting better data and more effective tools for preparing for and preventing public health threats.

## Abbreviations

AI: artificial intelligence
API: application programming interface
FAIR: Findability, Accessibility, Interoperability, and Reusability
HPO: Human Phenotype Ontology
*ICD*: *International Statistical Classification of Diseases and Related Health Problems*
*ICD-10*: *International Statistical Classification of Diseases and Related Health Problems Tenth Revision*
JSON-LD: JSON for Linked Data
LLM: large language model
ML: machine learning
MONDO: Mondo Disease Ontology
OSD: Open Syndrome Definition
OSI: Open Syndrome Initiative
PPHSN: Pacific Public Health Surveillance Network
SNOMED CT: Systematized Nomenclature of Medicine—Clinical Terms
SPIRES: Structured Prompt Interrogation and Recursive Extraction of Semantics
WHO: World Health Organization
